# Security and Privacy Analysis of Youth-Oriented Connected Devices

**DOI:** 10.3390/s22113967

**Published:** 2022-05-24

**Authors:** Sonia Solera-Cotanilla, Mario Vega-Barbas, Jaime Pérez, Gregorio López, Javier Matanza, Manuel Álvarez-Campana

**Affiliations:** 1 ETSI de Telecomunicación, Universidad Politécnica de Madrid, Av. Complutense 30, 28040 Madrid, Spain; mario.vega@upm.es (M.V.-B.); manuel.alvarez-campana@upm.es (M.Á.-C.); 2 Institute for Research in Technology, ICAI, Comillas Pontifical University, C. Alberto Aguilera 25, 28015 Madrid, Spain; jperezs@comillas.edu (J.P.); gllopez@comillas.edu (G.L.); jmatanza@comillas.edu (J.M.)

**Keywords:** connected devices, Internet of Things, privacy, security, vulnerability

## Abstract

Under the Internet of Things paradigm, the emergence and use of a wide variety of connected devices and personalized telematics services have proliferated recently. As a result, along with the penetration of these devices in our daily lives, the users’ security and privacy have been compromised due to some weaknesses in connected devices and underlying applications. This article focuses on analyzing the security and privacy of such devices to promote safe Internet use, especially by young people. First, the connected devices most used by the target group are classified, and an exhaustive analysis of the vulnerabilities that concern the user is performed. As a result, a set of differentiated security and privacy issues existing in the devices is identified. The study reveals that many of these vulnerabilities are related to the fact that device manufacturers often prioritize functionalities and services, leaving security aspects in the background. These companies even exploit the data linked to the use of these devices for various purposes, ignoring users’ privacy rights. This research aims to raise awareness of severe vulnerabilities in devices and to encourage users to use them correctly. Our results help other researchers address these issues with a more global perspective.

## 1. Introduction

Ubiquitous computing, pervasive technology, and ambient intelligence represent three enabling technologies that have greatly conditioned the advancement and development of the current Information Society (IS). These three Information and Communication Technologies (ICT) have mainly driven the development of the Internet of Things (IoT), which is a digital ecosystem of devices and everyday objects connected to each other without further human intervention [1]. Thus, the rise of the IoT has facilitated the creation of novel and more personalized ICT services.

The usefulness of these services and the IoT depends closely on their adoption in people’s daily lives, so it is based on their relationship with the most personal and intimate sphere of their users [2]. The higher this penetration, the higher the level of customization and usefulness of IoT services and applications. For this reason, this concept of an ecosystem of interconnected devices has had, in recent years, a significant impact on the consolidation of, e.g., telemedicine and electronic health services [3], industry 4.0 [4] or smart spaces such as Smart Cities, Smart Homes, etc. [5,6]. In addition, it has facilitated the creation of new market contexts such as toys and child development (the Internet of Toys) [7], the automobile industry (the Internet of Vehicles) [8], or even health (the Internet of Health Things) [9].

However, despite the clear benefits offered by the IoT paradigm, it is often plagued by security and privacy weaknesses of the underlying devices and applications. Since the quality and usefulness of IoT services depend on their incidence and penetration into people’s daily lives and on the use of sensitive user data, these weaknesses present a critical issue [10,11]. In particular, the GSMA IoT Security Assessments argues that manufacturers benefit more as this penetration increases. Therefore, the authors propose a good practice guide focused on proper design, development, and deployment to improve the security of these devices [12].

Related to this, in commercial IoT applications and services, most of these weaknesses are due to device manufacturers neglecting security to reduce the cost of their devices. In some cases, these low-cost devices present long life cycles, and they lack an ecosystem of cybersecurity developing patches for them, which may make them eventually vulnerable [13]. Furthermore, they are usually used by people who have not received training on good practices when using them (e.g., avoid using default passwords). Such human contribution makes them even more vulnerable, so studies such as [14] address this aspect to contribute to IoT security through practice guidelines for manufacturers, vendors, and users.

As a result, these devices have recently become the most attractive segment for cybercriminal organizations [15]. Based on the “Nokia Threat Intelligence Report 2020” [16], IoT devices are responsible for 32.72% of all infections observed in mobile networks in 2020. This matter is aligned with the growing number of IoT devices that are more and more connected to mobile networks. Moreover, they have taken part in the share associated with infected Android devices, which illustrates the shifting interest of the attackers towards IoT devices. Therefore, it is necessary to address the weaknesses of such devices from the early stages of development, paying special attention to the relationship of the technology with the social group at which they are directed. To this end, over the past few years, the aim is to standardize the security and privacy of connected devices through security and data protection provisions and standardizations [17] or laws such as the “Internet of Things Cybersecurity Improvement Act of 2020” [18].

Specifically, this study focuses on the social group made up of minors from 12 to 17 years old, who are considered digital natives, who avoid questioning the penetration of digital technology in everyday life. Throughout this document, we study the main security and privacy problems associated with the connected devices most used by young people.

A systematic literature review is carried out for this purpose, and the results are classified into a set of categories on which to analyze these vulnerabilities. After analyzing the vulnerabilities, problems, and security and privacy deficiencies of these devices, it is intended to offer a clear vision of the possible attack vectors or weak points of this technology, as well as provide training for youngsters on the existing risks and threats to the use of such technology and on good practices to avoid them.

The remainder of the document is organized as follows. Section 2 points out the background and scope of the study, focusing on connected devices used by minors. Section 3 sets out the method followed for the literature review of the research. Section 4 shows the literature analysis carried out for each of the categories of connected devices. For its part, Section 5 offers the results of the study and classification of security and privacy issues of connected devices. These results are discussed in Section 6. Finally, Section 7 offers the conclusion of the work carried out.

## 2. Background

As we have mentioned above, this report focuses on studying the penetration level of connected devices within the daily lives of minors, that is, people between 12 and 17 years old. Concretely, this study is oriented toward understanding the relationship between young people and any ICT device in the IoT ecosystem. To this purpose, we determined what types of connected devices are available to young people and how they integrate them into their daily lives, e.g., their preferred connected device, the different uses for a given device, etc.

Minors and young people from 12 to 17 years old are those born between 2003 and 2008, belonging to the generation known as Centennials or Generation Z [19]. This generation is the first to be considered entirely digital native since it is the first cohort to have the Internet at an early age, and they are highly dependent on new ICT technologies [20]. It also highlights their ability to adapt to permanent changes and go through enormous amounts of information, even though they sometimes find it difficult to discern between right and wrong. For this generation, the frontier between virtual and physical worlds has blurred, meaning that most of their interpersonal relationships are mediated by the Internet [21]. In this sense, over 90% of the European population (16 to 74 years) had access to the Internet in 2019, and at least 77% used the Internet daily [22]. These percentages considerably grow when we analyze the data regarding digital natives. Specifically, based on the UNICEF report “The State of the World’s Children 2017: Children in a Digital World” [23], children and adolescents under 18 already account for an estimated one in three Internet users around the world, and Eurostat estimates that 96% of adolescents (16 to 19 years old) use the Internet daily in Europe [24].

Focusing on minors’ Internet usage habits, the study presented in [25] points out they use it for various entertainment and leisure purposes, such as watching videos, accessing social networks, playing video games, etc. To a lesser extent, the Internet is also used to support them in completing school assignments, searching for related news, or making purchases. In this context of Internet habits and uses, the device most used by minors and adolescents is the smartphone, followed by smart TV and the desktop or laptop computer. In this sense, the study shows a large difference between the penetration/use of this type of connected device by minors depending on age, the use being greater as age increases. Tablets and video consoles have an incidence similar to the mentioned devices. On the other hand, smart toys and wearables occupy the least significant positions in terms of the preferred device for accessing Internet services, although their use has grown significantly in recent years. In addition, penetration rates have also been found for certain devices such as connected appliances, home security devices (alarm systems, smart locks, cameras, etc.), energy management systems (smart lights, smart plugs, smart thermostats, etc.), smart speakers or smart personal assistants, smart black/whiteboards, etc. [25,26].

According to this, the following categories of connected devices have been defined to perform the security and privacy vulnerability analysis presented in the next section:

1. Smartphones and tablets;
2. Smart TVs and game consoles;
3. Smart toys;
4. Wearables;
5. Smart home IoT devices;
6. Smart personal assistants;
7. Smart speakers;
8. Others, such as drones or cameras.

## 3. Method

### 3.1. Research Questions

A systematic literature analysis approach was followed to carry out the above-mentioned analysis. The initial search focused on determining the IoT-connected devices more used by the target population segment. To this end, the following research questions are posed:

RQ1. What is young people’s use of the connected devices they use the most?
RQ2. What are the main vulnerabilities associated with the use of these connected devices?
RQ3. What are the main security issues encountered with these connected devices?
RQ4. What are the main privacy issues encountered with these connected devices?

This literature review, led by these research questions, was concluded considering some of the main research databases. This study only evaluated references from 2017 onwards to guarantee a timely analysis, except for two references from 2015 and 2016, which were considered very relevant for the research study.

### 3.2. Databases

The research was carried out in the following databases, which focus on science and technology-related topics: ACM Digital Library, IEEE Xplore Digital Library, and ScienceDirect.

### 3.3. Search Terms

The search accomplished in these databases started by determining the terms to be considered in the search. Due to the large number of records obtained, these search terms were divided into two categories: (i) those referring to general aspects to be addressed in the research questions, and (ii) those referring to the different categories of connected devices.

- Common terms: “internet of things”, “children”, “vulnerability”, “security”, and “privacy”;
- Specific terms: “smartphone”, “tablet”, “smart tv”, “console”, “smart toy”, “wearable”, “smart home”, “smart personal assistant”, and “smart speaker”.

All terms have been included in the search query considering deviations from the word of interest, e.g., in the case of “smartwatch”, it has been assessed that “smart watch” appears. The resulting search query is as follows:

(“internet of things” OR “iot”) AND (“smartphone” OR “tablet” OR “smart tv” OR “console” OR “smart toy” OT “wearable” OR “smart home” OR “smart personal assistant” OR “smart speaker”) AND (“children” OR “vulnerability” OR “security” OR “privacy”)

### 3.4. Study Selection

The report selection process considered the following inclusion and exclusion criteria (IC and EC, respectively):

- (IC) Publications with specific information on the connected devices most used by young people;
- (IC) Publications with information on security and/or privacy vulnerabilities in these connected devices;
- (EC) Publications whose date is outside the range of interest: from 2017 to 2021;
- (EC) Publications that are not written in English;
- (EC) Publications with duplicated content;
- (EC) Publications with content outside the main objective of the literature analysis (based on title, abstract, and keywords);
- (EC) Publications with content outside the main objective of the literature analysis (based on the full content of the document).

To comply with the guarantee that the reports found included up-to-date and timely information, in the first search, those according to the first two EC were excluded, i.e., those whose publication date was not in the range 2017 to 2021 and those were not written in English.

After filtering those reports duplicated in databases, a scan was performed based on title, abstract, and keywords. This refers to the next two EC.

The resulting records, still labelled as possible reports for the final study, were evaluated at a higher level of detail, reading, in many cases, the full text. Thus, we excluded those studies that, although apparently of interest to the research, did not contribute relevant information to the literature review. In this way, the last EC and the two ICs were fulfilled.

### 3.5. Data Analysis

Samples resulting from the filtering were collected and analyzed at each stage of the process. With all the reports obtained, we conducted a mapping study to classify them into each research question. This exhaustive content analysis of the final selection of reports allowed us to classify these results and allocate them to each research question.

Whereas this literature review was concluded with rewarding answers to the research questions, it was complemented by a broader one with conferences and workshops, news, and technical reports from the industry. Although, initially, only references with publication dates from 2017 onwards were considered, two references were found, from 2015 and 2016, which do not meet that criterion but were considered particularly relevant to the research study because of the content they offered.

Considering all the above-mentioned criteria and phases, in an initial search, considering the range of publication dates of interest and the language in which they were written, 820 reports were obtained. After this first phase, those hundreds of reports that were duplicated in the databases were filtered out. Of the resulting reports, the information in the title, abstract, and keywords was evaluated to ensure that they were related to the proposed research questions; in this way, many reports that did not address issues related to our review of the literature were eliminated. The resulting 53 reports were evaluated in detail, and 13 of them were eliminated because they did not address specific security or privacy issues or did not provide any additional information regarding the others.

To complete the search, as mentioned above, other sources such as conferences, workshops, etc., were considered, from which 4 more documents of interest were included. A final search to complete the literature review included 2 more reports dated 2015 and 2016.

**Figure 1:**
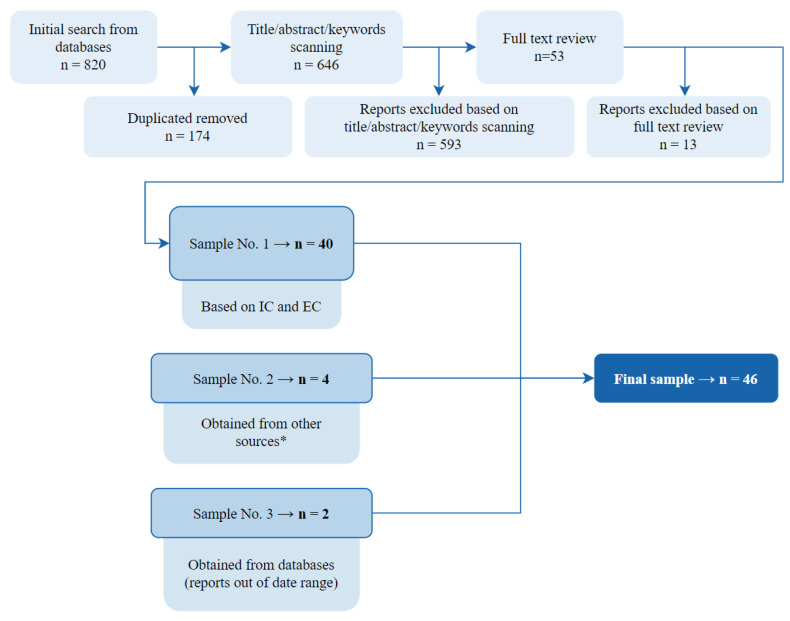
The selection process for the literature review. Those sources marked with * were extracted from conferences and workshops, news, and technical reports.

Hence, following the filtering process shown in Figure 1, the study finally considered 46 references. These references were classified considering the categories of devices most used by minors and were analyzed in detail, trying to identify common security and privacy issues. Thus, a glossary of security and privacy issues was defined. Finally, we assessed the relevance of each device category and each security and privacy issue based on the number of references. Thus, obtaining a traceability matrix by mapping device categories and the glossary of security and privacy issues.

## 4. Analysis of Vulnerabilities of the Connected Devices

As analyzed in [25], since digital natives almost exclusively conceive of their relationship with society only through the Internet and new technologies, they lack a clear awareness of the risks underlying the use of this technology. Furthermore, only a tiny percentage indicate that they carry out their activities online under the supervision or guardianship of an adult or following their recommendations. For this reason, it is essential to analyze the possible vulnerabilities associated with each type of connected device mentioned by studying and understanding the security and privacy risks associated with the use that minors and adolescents make of them.

### 4.1. Smartphones and Tablets

In terms of children’s use of tablets and smartphones, this study focuses on mobile applications, especially those targeted at minors, to narrow the scope of our research.

Mobile applications used by children may range from parental control apps (i.e., apps used by parents to monitor and limit their children’s mobile behavior) to mobile video games and other child-directed applications. Some initiatives, such as Google Play’s Designed for Families (DFF) program, aim to provide parents with a list of safe applications targeted at and suitable for children but may fail at properly estimating privacy compliance.

According to [27], some applications may circumvent the Android permissions system by making use of covert channels or side channels. For example, this research showed that, with enough permissions, applications could make use of the Secure Digital (SD) card as a covert channel to share the phone’s unique and identifiable International Mobile Equipment Identity (IMEI) with other unauthorized apps. Furthermore, some applications were found utilizing other channels to estimate and share user location through the device Media Access Control (MAC) address, Address Resolution Protocol (ARP) cache, or picture metadata.

Concerning parental control apps, the research work presented in [28] aimed to conduct an in-depth study of the Android parental control app’s ecosystem from a privacy and regulatory perspective, distinguishing between monitoring apps, which enable parents to monitor children’s behavior (including location), and restriction apps, which enable parents to filter content and to define usage rules to limit the children’s actions.

Regarding the use of permissions, the research conducted in [28] showed that parental control apps request 27 permissions on average, 9 of them being labeled as dangerous. In many cases, these dangerous permissions were used to leak data to remote servers. Most of these data leaks required logging user actions (e.g., logging a failed/successful authentication), and some involved sensitive data such as unique identifiers (e.g., the device’s IMEI).

Some of the apps analyzed in [28] also use custom permissions to obtain functionalities exposed by other apps’ developers, revealing (commercial) partnerships between them. A significant number of the calls to dangerous permission-protected methods were invoked only by embedded third-party libraries. Only half of the apps tested in this research informed users about their data collection and processing practices. A total of 59% of the apps admitted third-party usage of sensitive data, whereas only 24% disclosed the complete list of third parties embedded in the software.

Relating to regulatory compliance, Ref. [29] presented a framework for the automatic evaluation of the privacy behaviors of Android apps. The said framework was used to analyze the Children’s Online Privacy Protection Act (COPPA) compliance of 5855 of the most popular free children’s apps. The results of this analysis showed that a majority of the examined applications were potentially in violation of COPPA, mainly due to their use of third-party software. Several applications were found sending sensitive user information to remote servers, including geolocation data and the device owner’s email address and phone number. Moreover, the study found that more than half of the apps did not use a Transport Layer Security (TLS) in at least one transmission containing identifiers or other sensitive information.

### 4.2. Smart TVs and Game Consoles

Bearing in mind that, as mentioned above, one of the main habits of Internet use by minors is entertainment, there is a high penetration of smart TVs [30] and game consoles for these purposes.

Concerning the first one, smart TVs’ privacy and security issues arise because they are also connected to the Internet (and with other applications that require bank payment details for the purchase of products and content) and even have built-in cameras. In addition, they do not usually have systems against security threats, being vulnerable to intrusions. The need to study these aspects comes from many years ago. In March 2017, WikiLeaks announced that the Central Intelligence Agency (CIA) and UK Military Intelligence Section 5 (MI5) intercepted user information by installing malware on smart TVs [30]. Even at a Black Hat conference in 2013 [31], it was admitted that tests had been carried out to attack vulnerabilities in smart TVs by recording unauthorized content via their built-in cameras. Furthermore, the findings of [32] show how, using commercial Software Defined Radio (SDR) devices, it is possible to attack smart TVs using Hybrid Broadcast Broadband TV (HbbTV)—both by hijacking TV channels to impersonate the broadcasted content (channel injection attack) and by including the Uniform Resource Locator (URL) of a fake server in the HbbTV metadata (URL injection attack), which, in turn, may be used to commit social engineering attacks.

To ensure their safety, smart TVs are certified according to the Common Criteria (CC), a standard created by the International Organization for Standardization/International Electrotechnical Commission in 15408 to assess the safety and reliability of the device. In April 2017, the first CC EAL2 certification was proposed with LG electronics, including the analysis of non-basic vulnerabilities of smart TVs.

In other ways, due to the increased use of game consoles in recent years, the likelihood of cyber-attacks on them has also increased. This is because modern video game development tools interact with the Internet and third-party applications, which implies a greater personalized user experience but also greater data collections and damage if such data are compromised [33]. In addition, part of the video game ecosystem, notably the gaming cheat websites and modding forums, has been identified as a common pathway into cybercriminal activities [34]. The aforementioned cyber-attacks are focused on concrete game consoles with a great market impact and top games, such as World of Warcraft (WoW) or League of Legends (LoL). One of the attacks that caused significant financial losses and personal data theft was the PlayStation Network’s attack in 2011, with the suspension of service to its users for almost a month. These threats become even more dangerous when it comes to applications that require banking and credit card information.

According to a survey carried by a Spanish company specializing in cybersecurity S2 Grupo [35], more than 57% of video game users are unaware that their personal data can be attacked through their console and more than 85% ignore that the default security of the device can be modified and hardened. Therefore, users are highly exposed to these attacks, in part, because of this lack of privacy and security awareness about the game consoles.

According to [36], the most common types of attacks on game consoles are the following:

1. Distributed Denial of Service (DDoS) attacks to cause disruptions, as happened with the PlayStation Network in 2011 and XBOX Live in 2014;
2. Spoofed websites to obtain credentials, as happened with Twitch in 2014;
3. Stealing money with ransomware and scareware, as happened with many game consoles preventing users from playing their video games and demanding money in exchange for returning their game data in 2014;
4. Brute force attacks and keyloggers to spy on passwords, sometimes even carried out by other gamers;
5. Social engineering, such as phishing.

All these attacks are related to cybercrimes such as extortion, identity theft, fraud, child pornography, and information manipulation. Specifically, Ref. [37] conducted several tests against such attacks on the PS4 Slim, obtaining personal data such as names, user identifiers, personal emails, bank details, activity logs, etc.

### 4.3. Smart Toys

**Figure 2:**
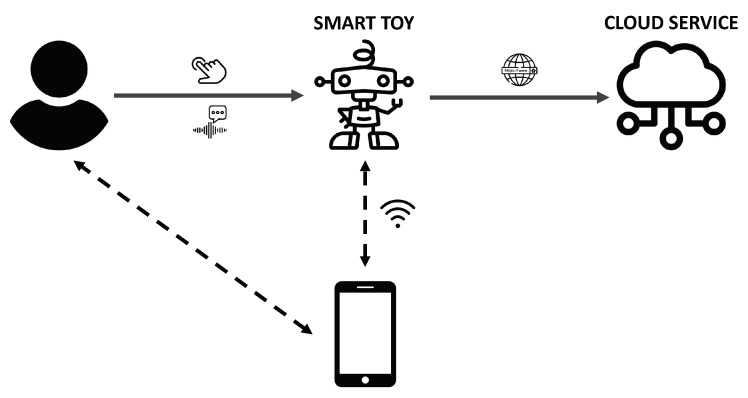
Example of interaction with a smart toy.

The smart toys industry is reaching more people every year. Recent studies, such as [38], show that revenues of the smart toys market will grow by 200% from 2018, achieving USD 18 billion in revenue in 2023. In this sense, smart toys are understood as toys that implement connection capabilities with different services, such as servers on the Internet or intermediate devices such as smartphones or tablets, as is shown in Figure 2. This family of devices uses their communication possibilities to share data between the device itself and the toy manufacturer or third parties’ servers to provide “intelligence” to the toy by applying data processing techniques with the information collected from the user. In order to collect information about the user to provide their functionality, the toys have several sensors, cameras and microphones incorporated.

The data collected by the toy and the processing applied range depending on the category and functionalities of the toy. For example, a recent security analysis of eleven smart toys [39] shows that all the toys studied collected personally identifiable information, and nine out of eleven shared this information with one or more third-party servers for ads and analytic purposes.

Since the target market is children, this ecosystem of products should be extremely careful about the information collected and its treatment and protection. Unfortunately, the entire group of smart toys analyzed in [39] suffered from several security vulnerabilities that directly affect the confidentiality of the data collected from the children and the integrity of the toys.

Regarding specific studies about some smart toy models, the study [40] reveals that Dino by CogniToys can be attacked to eavesdrop and decrypt the network traffic between the toy and the Cloud, which allows a malicious actor to listen to the recorded voice messages. The vulnerabilities present in the device also enable an attacker to remotely inject audio (malicious messages) into a unit, putting at risk the safety of children. In another analysis centered on the Fisher-Price SmartToy Bear [41], it was discovered that the user has no means to disable the built-in camera or even detect if it is active. Furthermore, an attacker can physically manipulate the toy since there is no access control to interact with its underlying operative system. This situation enables a malicious actor to modify the behavior of the toy, turning it into a spying device that can be accessed remotely anytime once modified. Finally, the research [42] focuses on three connected devices geared toward kids and teens. Specifically, security and privacy vulnerabilities of the application and the communications network associated with a hydration bottle for children, a pet, and a fitness band are analyzed. The vulnerabilities detected included a lack of encryption of the data sent (between the devices, the application, and the server), lack of authentication to access personally identifiable information, etc. Moreover, the study concludes that such vulnerabilities violate both COPPA and the privacy policies of each connected device.

Because of these attacks, Ref. [43] proposed a classification of toys according to the privacy and security they offer to protect children’s personal data. To this end, ChildShield provides privacy and security labels to inform about how the smart toy processes the user’s data.

### 4.4. Wearables

Wearable technology refers to everyday accessories or clothes improved by electronics to provide them with processing and communication capacity. It receives different names depending on the field of study, work context, or the market niche where it is included. Some examples include fashion technology or fashion electronics, smart textiles, smart wear, skin electronics, or simply wearable devices. Some of the most important examples are the smartwatch and activity tracker [44].

The current success of wearables is primarily due to the acceptance and adoption of the base element they emulate (e.g., watches, footwear, etc.). This high level of acceptance has facilitated their inclusion in people’s daily lives, favoring their application to eHealth, sports and personal care, personal safety and location, or even entertainment. In the case of children, wearables are a personal safety tool introduced by their parents, which allows for tracking the location of minors at any time. For its part, adolescents use these connected devices as a new sports training accessory. However, the adoption of wearables is very far from that of other connected devices such as smart speakers, game consoles, etc. [21,25], although it has grown in the last five years [45].

**Figure 3:**
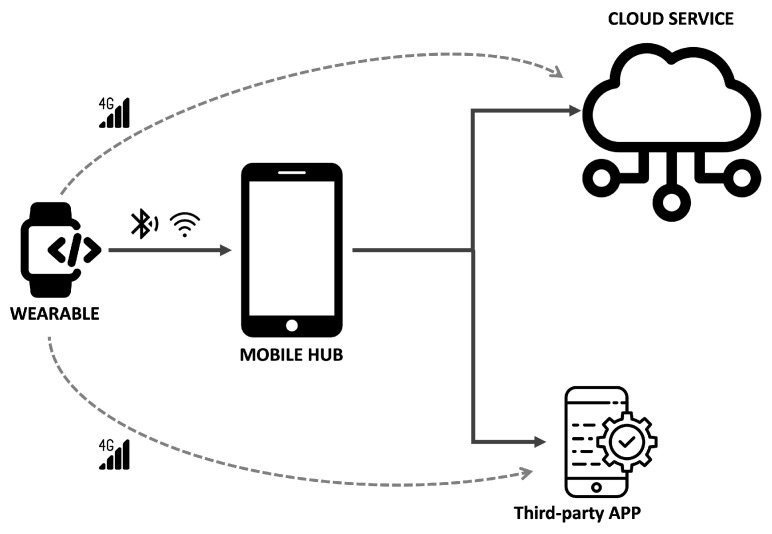
Wearable communication overview.

In general, the integration of these connected devices is carried out through wireless communication (Wi-Fi, cellular, Bluetooth, etc.) with other more powerful devices, typically smartphones or servers, and this is where most of the security and privacy threats appear [44] (Figure 3). This connection arises from the fact that wearables acquire data that must eventually be transferred to another element (device or server) with a greater computing capacity and that offers the underlying service. This information is usually sensitive (clinical data, location, etc.) and therefore susceptible to being compromised [46]. Figure 3 sketches the relationship between wearable devices and the other elements that make up an IoT service.

In this sense, the research work presented in [47] analyzed the information transferred between different wearables (Android Wear, watchOS, and Tizen) and host devices (smartphones) when performing a pairing. The results show a high transfer of sensitive data between smartwatches and host devices with light or no notification to the user. Additionally, it was found that users typically use the same credentials for both devices. In [48], the authors presented a methodology to perform a vulnerability assessment of wearable devices involving Reconnaissance, Scanning, Enumeration, and Penetration phases. The said vulnerability assessment was then used to analyze and identify the security issues of three different wearable devices that communicate via Bluetooth Low Energy (BLE) with a smartphone: Fitbit Charge and Fitbit Alta by Fitbit, and Easy-Fit by Cellular Line. The analysis results show that the principal vulnerabilities affecting these technologies are weak methods for generating Short Term encryption Keys (STK), such as the Just Work or Passkey Entry methods, and the lack of a pairing and bonding process between devices and smartphones. Both the Easy-Fit and Fitbit Charge present issues regarding these vulnerabilities, allowing a potentially malicious agent to sniff sensitive user information exchanged between device and smartphone.

In a broader sense, the work presented in [44] provided a comprehensive survey and classification of commercially available wearable devices, the communication security threats they face, and solutions to these issues available in the literature. The study analyzed the threats regarding their effect on the confidentiality, integrity, or availability of the information handled.

In terms of threats to confidentiality, most wearable devices were vulnerable to eavesdropping, traffic analysis, and information gathering attacks due to the use of BLE as a means of communication. Most eavesdropping and traffic analysis attacks were related to improper implementations of the BLE advertisement process or the use of static device addresses. In contrast, information gathering attacks tended to involve breaking the key exchange in BLE pairing or collecting information about other user devices, such as smartphones [49].

Although not as common as threats to confidentiality, three types of attacks were identified concerning threats to integrity: modification attacks, replay attacks, and masquerading attacks (in which the attacker impersonates an authenticated device to steal data or inject false information into the system). All the vulnerabilities found in this context were due to the lack of authentication methods or the absence of encryption in communications between wearable devices.

In terms of availability threats, DoS attacks were found to be the primary type of attack against wearables. As with the other threats described, availability attacks were only possible due to manufacturers’ implementation deficiencies.

As commented above, the authors of [44] also elaborated on proposed solutions to the threats identified. When it comes to wearables, most devices implement some form of MAC layer encryption to protect confidentiality and integrity. This may not be sufficient in most cases, but in some devices (especially in more compact wearables, such as smart earbuds), resource limitations such as memory and power do not allow advanced encryption methods to be implemented. In this regard, there are several solutions for low-power encryption protocols and key exchange mechanisms. Apart from encryption, authentication protocols also proved to be effective in protecting the integrity, but not much work was found to ensure availability, as threats to the availability of wearables are limited.

In the case of smartwatches for children, the research work presented in [50] analyses the security and privacy concerns of six smartwatches for children and their corresponding server platforms. The study discovered severe security vulnerabilities that allowed to take over the devices through standard attack techniques. By lacking a strong policy of encryption of the communications between devices and back-end platforms as well as vulnerabilities related to the authentication process, most of the analyzed devices allow hackers to access sensitive data, conduct illegal remote surveillance of the user, or spoof the communications from and to the smartwatch. In addition, most of them send the gathered data to servers and back-end platforms located outside the EU (e.g., China). In this way, the report [51] from the Norwegian Consumer Council corroborated the ease of access to information shared by this type of wearable. Specifically, it analyzed the privacy vulnerability associated with how these devices share GPS location information.

### 4.5. Smart Home IoT Devices

The smart home has taken a leading role within the IoT ecosystem in recent years. In order to transform our home into a more comfortable and efficient place, control techniques and automation of tasks can be applied to enhance the quality of life of the inhabitants [52]. Thus, the smart home is understood as a particular case of smart space where ambient intelligence and ubiquitous computing transform a non-technical space into an environment capable of adapting to the needs and tastes of its inhabitants. Such a transformation takes place thanks to the IoT, as it is the link (through interaction models based on affordability) between the physical space (home-side) and the enhanced virtual world (smart-side) [53].

In this sense, many smart home IoT devices allow inhabitants to easily control everything, from temperature or lights to the security perimeter (by complex home security systems). However, integrating such devices into a home can pose a serious risk to security and privacy. That is why researching privacy and security vulnerabilities associated with smart home IoT devices is key to preventing data privacy from being exposed. The most common attacks related to these devices are due to a lack of data encryption [54,55,56,57], allowing attackers to gain access to personal data through vulnerabilities in the device itself, e.g., by using the well-known Man in the Middle (MitM) attack.

**Figure 4:**
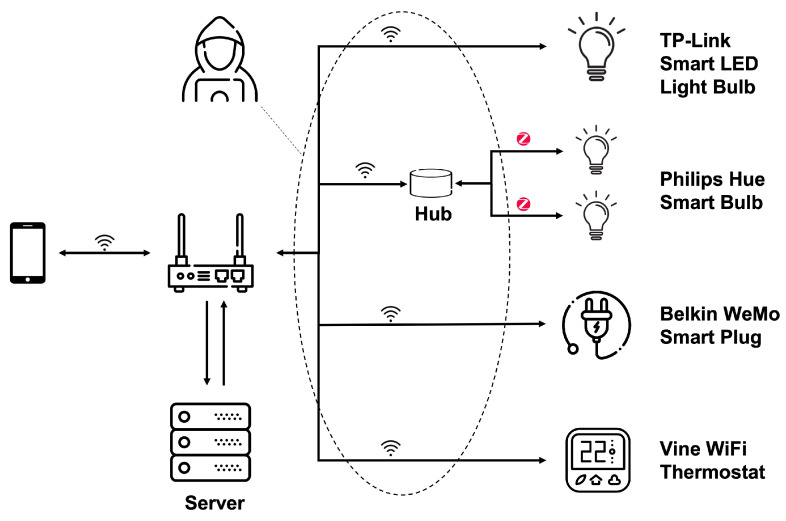
Smart home IoT devices communication scheme.

The research work presented in [54,57] analyses a set of attacks seeking to extract sensitive user data. First, they analyzed the specific characteristics of devices that leave data exposed to threats, such as the device itself not encrypting the transmitted data or leaving data visible in plain text. Afterward, they evaluated MitM and replay attacks to check which devices were vulnerable to them. All these vulnerabilities are evaluated on several models of light bulbs, Wi-Fi thermostats, fire and flood prevention alerts devices, smart plugs, and two smart personal assistant models, the Amazon Echo and Google Home Mini. Among all the devices studied, light bulbs, plugs, and thermostats are the most vulnerable. Specifically, TP-Link’s smart LED bulb, Philips Hue smart bulb, Belkin WeMo smart plug, and Vine Wi-Fi thermostat have privacy vulnerabilities due to the lack of encryption of data transmitted through the communications scheme shown in Figure 4. The remaining models do not allow an attacker to obtain information by the means of studies in this report.

In the case of [55], authors studied the problems associated with the leakage of personal information through smart home devices themselves and those related to data security and privacy due to a lack of information provided to users. That is a problem due to a lack of awareness of the risks associated with the use of certain devices. Specifically, these vulnerabilities are studied in a smart TV, smart speaker, smart plug, IP camera, smartphone, smart car, and other devices within reach of the user in their daily lives. Some of the main threats found in them are unauthorized collection and distribution of data to third parties, remote control of these devices (temperature, lighting, etc.), insecure communication protocols, lack of device authentication, and a weak password policy.

Along this very same line, Ref. [58] analyses a commercial smart home IoT platform from a security perspective. First, the main components of the platforms are identified, obtaining an architecture very similar to the one shown in Figure 4, composed of: (1) sensors and actuators, (2) a home hub that is connected to both such sensors and actuators, and to (3) the Cloud of the manufacturer, and (4) the mobile or web apps that allow controlling such sensors and actuators remotely through the Cloud. The study reveals several security flaws, such as weak or lack of authentication to perform sensitive tasks such as saving a system configuration backup in the Cloud or lack of encryption in some message exchanges (e.g., after sensing a wrong username). In addition, this work also illustrates how commercial Software Defined Radio (SDR) devices can be used to perform replay attacks that allow directly controlling the home sensors and actuators, bypassing the application provided by the manufacturer.

The research conducted in [56] studies the possible vulnerabilities in a system based on smart plugs controlled by the EdiPlug application installed on an iPad. The attacks that are of interest, in this case, are the following:

- Device scanning attack: it is shown that the MAC addresses of the Smart plugs are left unprotected;
- Brute-force attack: This attack is carried out if the user has changed the default passwords;
- Spoofing attack: this attack attempts to obtain the controller’s credentials using a software bot that impersonates a socket and authenticates with the remote controller;
- Firmware attack: it consists of installing malicious firmware to control the smart plugs remotely.

In all these espionage attacks, the attacker gains access to the user’s personal information (e.g., authentication credentials) due to the lack of authentication, either by capturing data transmitted between devices or even by taking full control of the device remotely.

As highlighted in [59], most of the attacks mentioned above can be mitigated by implementing a secure and adaptative hub that, through a publish–subscribe architecture, communicates with each smart home device through a secure communications channel. It also implements more secure authentication mechanisms, such as the Advanced Encryption Standard 256 (AES256). The testbed of this research is composed of a set of smart home devices, such as Philips Hue Lamps, TP-Link NC200 camera, Samsung Smart Things hub, and LG smart TV.

Finally, a vulnerability has been recently reported by Romanian cybersecurity technology company Bitdefender in the widely used ITEAD Sonoff/eWeLink platform. Notably, such a cloud-based vulnerability would allow taking over any device remotely by guessing or brute-forcing its unique identifier [60].

### 4.6. Smart Personal Assistants

**Figure 5:**
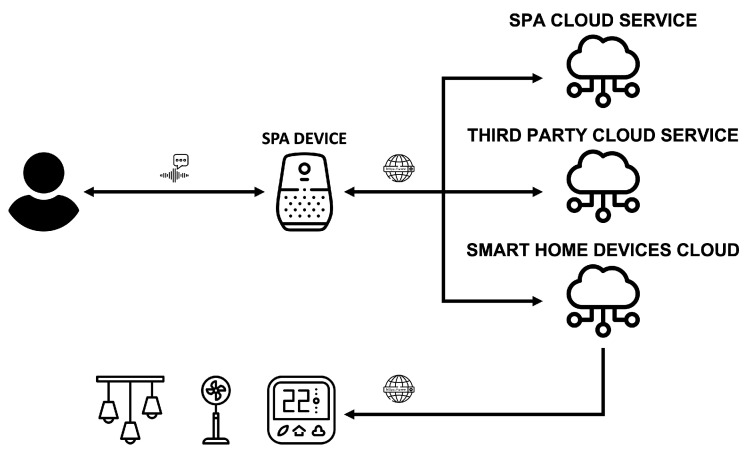
Smart Personal Assistants environment.

A Smart Personal Assistant (SPA) or Intelligent Personal Assistant (IPA) is a software agent that offers a humanized interface between computer systems and their users, implementing a person–system interaction model based on voice. The user interacts with the system by issuing voice commands to a device with microphones, e.g., a smart speaker or a smartphone, and this, in turn, interacts with the SPA cloud service or any other third-party cloud service, as Figure 5 illustrates.

The commands are sent and processed in the Cloud, where the user’s intention is extracted and returns a response that executes the required instruction. Examples of this type of device or technology are Apple’s Siri, Google Assistant, Amazon Alexa, or MS Cortana.

According to a recent survey about the security and privacy in the SPA environment [61], due to their architecture, design, and lack of several controls, vulnerabilities arise in five different fields:

- Weak authentication;
- Weak authorization;
- Profiling;
- Adversarial Machine Learning (ML);
- Underlying and integrated technologies.

Some of the vulnerabilities presented below are exploited in the research [62] using two proof-of-concept attacks to demonstrate the harm a malicious actor could cause. Since the SPA does not authenticate the user, an attacker can perform a home burglary after unlocking the access door by asking the SPA to open it. Moreover, a malicious third party can also submit a fake order at Amazon.com on behalf of the user just by issuing the correct command to the SPA.

The research work presented in [63] explores more security and privacy issues related to the SPA. Due to the lack of user authentication when communicating with the SPA, the attacker can maliciously alter the commands given by the user through third-party skills. This research is focused on voice-based remote attacks on Alexa and Google Assistant, which collect private information through user–system conversations:

- Voice squatting attack (VSA): this threat consists of taking advantage of the phonetic similarity of certain words to activate one of the malicious skills mentioned (unwanted by the user who gives the command);
- Voice masquerading attack (VMA): this threat consists of not complying with switching/completing tasks, but the SPA continues acting without the user being aware of it;
- This study is also focused on mitigating the threats mentioned. To this purpose, techniques are developed to mitigate VSA and VMA attacks. These techniques are based on the length of the invoked commands (phonetic distances) so that, by identifying if they are phonetically similar, squatting attacks can be avoided.

Several techniques prevent an attacker from impersonating the system (masquerading attacks) by identifying expressions such as those used by the user. To this end, the study makes use of natural language processing (NLP) and machine learning. In addition, the research supports a survey of Amazon Echo and Google Home users to understand how they interact with these devices. One example given in the article is a VSA based on a phishing attack using a fake credit card expiry notice to get the user to reveal his real account credentials.

Finally, the work carried out in [64] shows how it is possible to develop voice commands inaudible for the human being but yes for the assistants. In this way, through what they called a dolphin attack, they sent and executed hidden (inaudible) commands to different SPAs. In conclusion, they offered solutions both at the hardware and software level that allow mitigating this vulnerability.

### 4.7. Smart Speakers

A smart speaker is a type of speaker with interactive voice-based capabilities, mainly related to the previously mentioned SPAs [65]. These connected devices represent a hub element for the digital home since it allows communication via Wi-Fi or Bluetooth with a multitude of connected devices. Wireless speakers have also been included in this category as they are connected devices with significant penetration among the adolescent population (although they do not have computing capacity as such).

**Figure 6:**
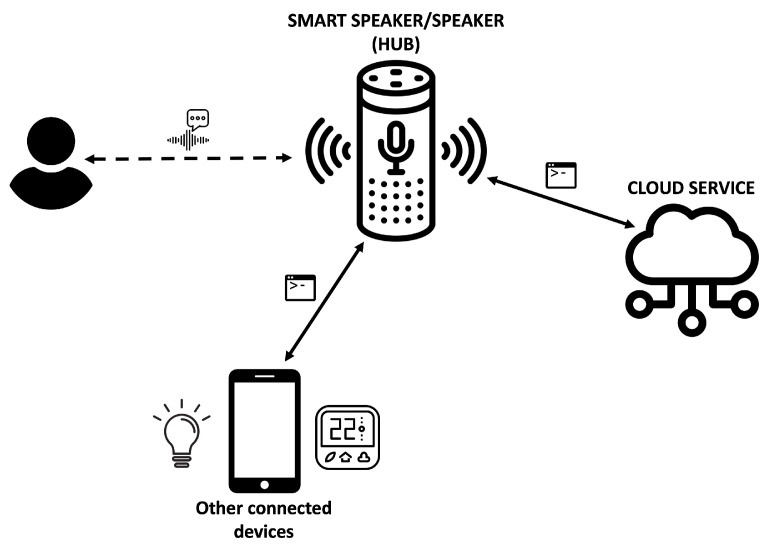
Smart speaker communication overview.

As Figure 6 shows, a voice interaction environment based on a smart speaker offers two communication ways: one based on voice commands and the other based on machine commands [65,66]. In this sense, the smart speaker should transform voice commands coming from the user into machine commands or orders to be transmitted to a cloud service (e.g., voice assistants) or to another connected device, as well as a transform machine command coming from service/system-side into voice messages to inform the user. Therefore, the smart speaker acts as a communication hub, providing several communication interfaces to the environment. These two ways of communications (voice and machine commands), together with the network interfaces supported by the smart speaker (Wi-Fi and Bluetooth), represent the main attack vectors for this connected device [67].

The literature analysis shows a change in the focus of the attacks perpetrated on this type of connected device. Initially, the attacks were aimed at exploiting the API vulnerabilities offered by the device in the same way that a web server or a computer is attacked [67,68]. Now, this attack approach has been redirected towards two specific targets: the communication interface used by the device (mainly Bluetooth) [69,70] and the voice-based interaction model [71,72,73].

In the case of speakers (and some smart speakers) that use Bluetooth to connect with other connected devices, the attacks suffered focus on exploiting how the pairing key is negotiated. This attack, called Key Negotiation of Bluetooth (KNOB), directly affects the standard, so all devices with a version lower than Bluetooth 5.1. are susceptible to being affected [69]. Some researchers have focused on verifying what effects this attack can have in the specific case of Bluetooth speakers. In this way, Ref. [69] found that attackers can gain complete control over a Bluetooth device without alerting the user or carrying out more harmful attacks such as monitoring a conversation. For its part, Ref. [70] showed how to attack the Transmission Control Protocol (TCP) communication between the device and the related server. In this case, using machine learning techniques, researchers managed to extract private (even encrypted) information sent from an Amazon Echo to Amazon’s servers with an accuracy of 97%.

Recent advances in deep learning techniques have favored the proliferation of attacks focused on the voice-based interaction model implemented by smart speakers, such as VSA [72]. In this sense, the authors discuss the vulnerability of the Amazon Echo and Google Home smart speakers to encrypted traffic analysis attacks (voice command fingerprinting). Using deep learning techniques, they developed a system capable of generating fake voice commands, managing to correctly infer simulated voice commands on encrypted traffic with an accuracy of 92.89% over Amazon Echo. Furthermore, authors in [71] studied the effects of side-channel attacks on smart speakers and proposed a solution to mitigate the loss of sensitive information associated with this vulnerability. Finally, the authors of [73] proposed the use of audio generating devices (TV, Radio, other speakers, etc.) to implement an attack called remote voice control or REEVE. Through this attack approach, they managed to compromise a smart speaker (Amazon Echo) to access some associated applications (Alexa skills and “If This, Then That” applets) and the devices to which it is connected. As in the rest of the articles analyzed, the authors proposed the corresponding countermeasures to mitigate this type of attack.

Lastly, research on privacy and smart speakers has focused mainly on analyzing user behavior and understanding the user’s perception regarding the connected device [74,75,76]. In [76], the authors attempt to understand user awareness of the implications of living with this type of device. For their part, the works [74,75] focused on analyzing users’ perceptions of smart speakers. From these studies, there is an inconsistency between users’ behaviors and their privacy perception when interacting with these devices. For instance, it has been observed that users behave in a relaxed manner, from a security point of view, whereas their concern for controlling the information captured (heard) by smart speakers increases.

### 4.8. Others

Other types of connected devices may not be so widely used by the target population segment but deserve to be considered for the comprehensiveness of this analysis. Some relevant devices within this category are drones, cameras, and intimate devices. Regarding drones, although their use is subject to regulation, for some time now, they have become a familiar toy for teenagers interested in technology. However, security flaws have been found and reported in commercial products that may involve a risk for their users and their surroundings. The research conducted in [77] analyses the security of some drone models manufactured by Parrots. This research shows that by applying reverse engineering to the binary protocol used between the pilot and the drone, an attacker can understand all the commands, allowing an attacker to inject such commands and hijack the drone, going unnoticed by the legitimate user. In addition, this work also reports a vulnerability in the application, which allows locating these drones worldwide.

Regarding IP cameras, research conducted in [78,79] discusses some security vulnerabilities in this kind of device. Although they may not be widely used by the target population segment of this study, they can help to configure this kind of device properly, thus avoiding potential problems such as the Dyn attack in late 2016, which was an orchestrated DDoS attack using IP cameras and similar devices infected with the Mirai malware due to the use of weak or default passwords. This incident illustrates that compromised IoT devices represent a severe risk to the systems where they are integrated, their users’ data, and third parties [80]. This incident led pioneer states in the US such as California or Oregon to develop specific regulations for IoT security by the beginning of 2020.

Finally, the interest of the target population segment in intimate devices is unclear. Although many minors in the considered age range are already aware and interested in sexual activities, no data have been found regarding if this kind of device catches their attention. However, these devices may entail security, privacy, and even safety concerns, and the information they manage is so sensitive that it makes them relevant in any case [79]. As a matter of fact, if an intimate device that can be controlled remotely were hijacked, this would constitute a form of sexual abuse. Likewise, the data collected by intimate devices might represent a top target for stalkers. Report [79] analyses two intimate devices, namely Vibease and OhMiBod, and in both cases, vulnerabilities are found. In the case of Vibease, it was discovered that the Android app exchanges messages without any encryption but just encodes them using base64. As a result, the user credentials can be compromised, chat messages could be intercepted, and the legitimate user could even be impersonated. In the case of OhMiBod, an attacker can also impersonate a remote user and send commands to the device by exploiting an authentication vulnerability found in the Application Programming Interface (API) server.

## 5. Results

**Figure 7:**
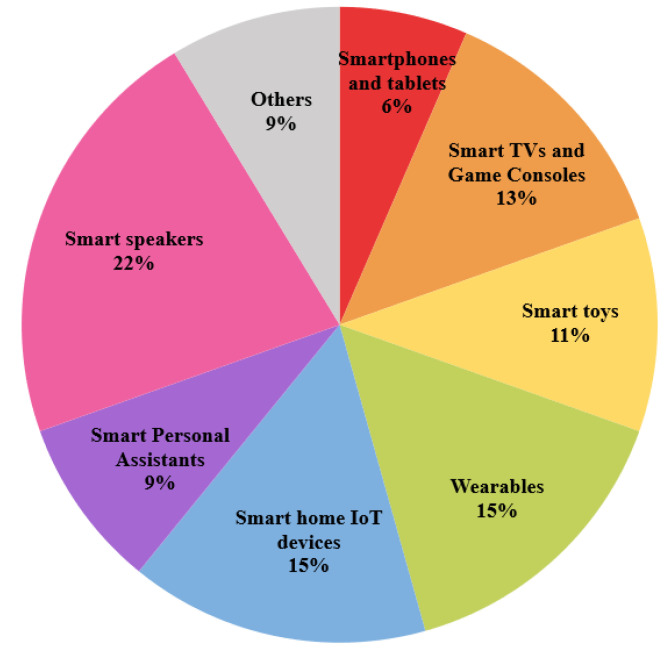
Distribution of security and privacy reports analyzed.

The analysis of security and privacy issues of connected devices presented in the previous section has considered about 46 references from a total of 59 research works (mainly academic but also including journalistic works). References dated in 2016 or earlier have been excluded from the study to provide an accurate and up-to-date view of the research context, except for two references from 2015 and 2016, which were considered particularly relevant to the research study. From the initial set obtained, 13 references were eliminated from the final analysis set because they did not address specific security or privacy problems of connected devices or did not offer more information than detailed in the rest of the articles. Thus, and considering the classification of connected devices presented in Section 3, Figure 7 shows the distribution of references studied by the type of device categorized.

As can be seen, smart speakers are the device category with the highest incidence within the study (22%). Considering SPAs and Smart speakers usually work together, the mashup formed by both categories represents 31% of the reports analyzed, an incidence much higher than that of the rest of the categories. Smart home IoT devices, wearables, smart TVs, and game consoles are the following most important categories with an incidence of around 13–15%. For its part, the smart toys category acquires an incipient role with an 11% incidence. In case of the “Others” category, it encompasses three recent studies about devices that, although minors and young people do not widely use them, do have an impact on considered (9% of the total) and interesting results on devices with embedded cameras (baby monitors, drones, etc.) or they affect sensitive aspects of users. Finally, with the lowest incidence of all device categories, we found that for the smartphones and tablets, the studies on security and privacy collected and analyzed have been reduced to an impact of 6% because, although they are the preferred devices and most used by young people, this document has been only focused on the most popular applications used instead of on technology per se.

In this way, the study’s main result is a classification of the security and privacy problems analyzed in 10 different groups, 7 referring to security and 3 to privacy issues. Table 1 shows this classification, providing an identifier and complete definition of each privacy or security issue and some related cybersecurity terms (vulnerabilities, attacks, risks) that have been analyzed in the reports. This identifier is helpful for the traceability matrix that relates each privacy or security issue to each type of device. Each of the identified issues refers to security vulnerabilities, attacks, or privacy risks. For example, all the vulnerabilities and attacks detected in the reports analyzed using the voice (natural or processed) as a means of action have been grouped into the same category: uncontrolled voice interaction. This security issue considers problems related to VMA, VSA, and hidden voice commands. Other examples included in the classified security issues are Structured Query Language (SQL) attacks within the code injection problem, MitM attacks related to data interception, or DDoS attacks related to the takeover.

Based on this categorization of security and privacy problems, a traceability matrix has been generated relating these to each type of device. This matrix is shown in Table 2.

**Figure 8:**
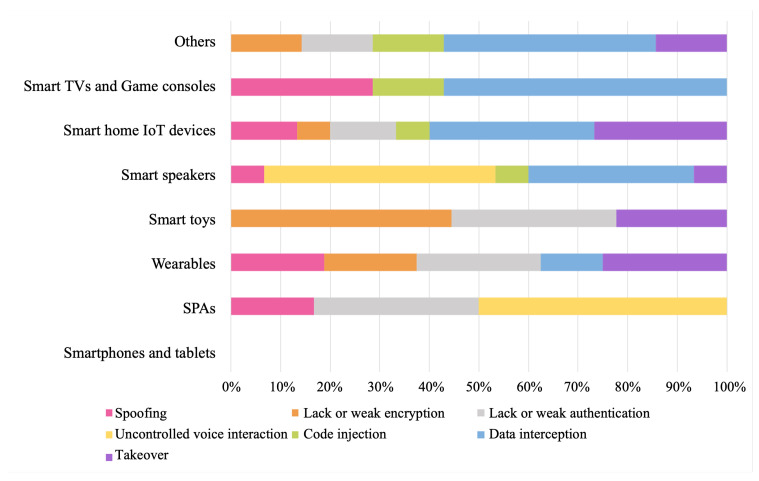
Security issues per connected device category.

**Figure 9:**
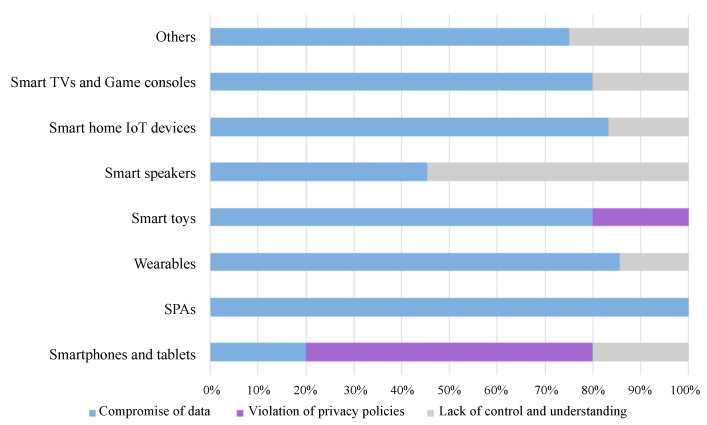
Privacy issues per connected device category.

In addition, Figure 8 and Figure 9 show the incidence of each security and privacy issue, respectively, on each type of connected device. This incidence has been determined from the number of reports that refer to the type of device in question and the problems addressed. As can be seen in Figure 8, the category most affected by security problems, according to the literature analysis, is smart home IoT devices with six of seven issues. Next are wearables, smart speakers, and other devices (because the latter category includes a mix of devices related to various issues) with five of seven issues. The rest of the categories have been affected by three of the seven defined security issues. For its part, Figure 9 shows us that all the categories, through the security problems detected, allow user data to be compromised. In addition, the articles analyzed indicate that users of all categories, except smart toys, show doubts or fears about the management that devices make of personal data. Finally, only articles oriented to the analysis of applications or devices specially designed for minors (smart toys and smartphones, and tablets) detected violations of privacy laws, specifically COPPA.

**Figure 10:**
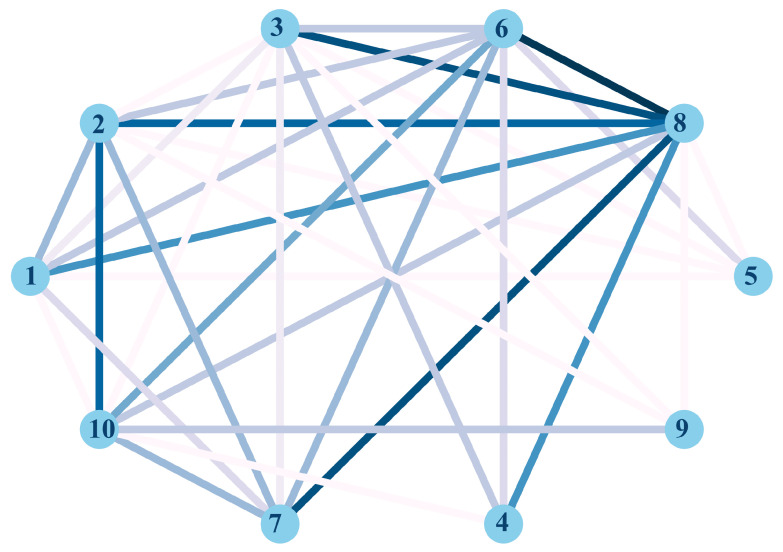
Relationship between all security and privacy issues defined. The identifier numbers in the figure refer to security and privacy issues: (1) spoofing, (2) lack of or weak encryption, (3) lack of or weak authentication, (4) uncontrolled voice interaction, (5) code injection, (6) data interception, (7) takeover, (8) user data being compromised, (9) violation of privacy laws, and (10) lack of control and understanding.

Following the study, the relationship between the defined security and privacy issues has been evaluated (Figure 10). Firstly, security issues such as a takeover, inadequate management of authentication and encryption, and data interception are the main factors behind compromising the information managed by the connected devices considered in the study.

Secondly, the violation of privacy policies (COPPA in this case) leads to a perception of loss of control and understanding of how data are managed from a privacy perspective. This issue is mainly conditioned by security problems related to data encryption. In this sense, the possibility of intercepting data also negatively influences this perception.

Another remarkable fact is that the security problems related to the possibility of uncontrolled voice interactions are conditioned to the maturity and quality of the device’s authentication system. Furthermore, this problem is related to the possibility of intercepting voice data broadcast by users. On the other hand, spoofing in connected devices is conditioned, mainly, to the level of data encryption and, to a lesser extent, to the implemented authentication system. Indeed, a real spoofing problem generally involves data interception. Finally, it has been shown that code injection, in general, is used for data interception, although it has a certain relationship, as expected, with spoofing problems.

## 6. Discussion

The results obtained in this work are based on the analysis of vulnerabilities of the connected devices most used by the target group. To achieve this, we first followed the exposed method based on a literature analysis starting from a previous categorization of connected devices. As a result of the literature analysis, Figure 7 shows the percentage distribution of the research papers studied based on this categorization of the types of devices most used by young people. We can see that all types of devices are highly interrelated, so many of the reports that were found discuss security and privacy issues focused on more than one device category. Undoubtedly, the devices most used by young people are smartphones and tablets. However, in this review, we have focused on those reports that offer security and privacy information concerning extended use applications since these are the ones where they spend more time when using the device. The devices for which more reports about vulnerabilities have been found are smart speakers due to their high adoption in the lives of young people in recent years, making them attractive to an attacker. These have been analyzed in conjunction with SPAs as they commonly work together, making them highly related to each other.

On the other hand, we highlight the number of devices they have at their fingertips in their immediate environment and continuously in their daily lives: from any smart home IoT device, such as sockets and light bulbs, to wearables, smart TVs, and game consoles. In these categories, we have assessed the incidence of these devices and how they use them to understand the potential security and privacy leaks associated with their use. Finally, with a similar incidence, we find smart toys and other connected devices such as webcams, drones, or intimate devices.

Considering the results presented previously, the RQ1 has been addressed as this analysis provides information on the use that young people between 12 and 17 years old make of the connected devices available to them. However, multiple limitations were encountered in addressing this research question due to the lack of related documentation. Therefore, we consider exploiting this line of research in-depth to gather more information from that obtained with specific and updated data for RQ1.

Once the literature analysis has been performed, the vulnerabilities of the connected devices are studied. The results of this process involve the classification of these vulnerabilities in a glossary of security and privacy issues for each category, as Table 1 shows, and to contrast this information, the results are included in the matrix, shown in Table 2.

These results are also shown through two graphs showing the impact of each security and privacy issue for each type of device categorized, Figure 8 and Figure 9. In them, we can see that the security issues that stand out from the rest are lacking or weak authentication and encryption methods. On the other hand, we highlight data interception; however, this type of attack is not found in smart speakers as it does not perform authentication on the voice channel when the user gives a command. For the same reason, we found an uncontrolled voice interaction predominant between smart personal assistants and smart speakers. Moreover, with a similar incidence, we found problems of takeover, spoofing, and, to a lesser extent, code injection, which are closely related to the privacy offered by the connected devices.

Concerning the latter, the study has identified three main types of privacy issues in these devices. The first of these problems is the compromise of user data, either through loss, misuse, or unauthorized access to it. The second one is the lack of user control and understanding of the dangers associated with the use of these devices. Finally, some reports have exposed violations of privacy laws such as COPPA. These problems are of particular concern when dealing with minor-age users. In this case, it is necessary to apply laws such as COPPA to protect minors’ data in a clear, complete, and understandable way for the user. That issue is why this privacy problem stands out mainly in smart toys, smartphones, and tables since they are the devices that are used by minors at an early age.

However, the fact that certain privacy problems are not explicitly mentioned in some of the categories does not imply that this vulnerability cannot occur, but rather, it does not stand out compared to others. In fact, in one way or another, the privacy issues highlighted are interrelated. For example, if a user’s personal data are compromised, it would also violate the user’s privacy as regulated in the privacy policies.

In terms of privacy, to avoid all these problems, it is not only advisable but necessary that the user knows and controls the use that the devices make of their data. To this end, understandable information adapted to all users must be provided. In general, there are multiple studies that address security and privacy issues in the IoT domain from a different perspective. This is the case of [81], the authors address the challenge of security in massive data processing in IoT devices in the field of intelligent healthcare. Their proposal shows possible solutions that improve the security of devices by using blockchain, ensuring confidentiality, privacy and data integrity. Other solutions, such as those exposed by [82,83], focus on improving device authentication protocols and monitoring the data flow of IoT devices in order to avoid security and privacy leaks in network traffic.

Our research and relevant results provide answers to research questions RQ2, RQ3, and RQ4. Table 2 summarizes these results in a matrix of security and privacy issues found for each device category studied.

Considering our analysis and those existing in the literature, we propose three main lines of research as future work. First, due to the absence of literature on this aspect, we intend to continue with the line of research on which devices are most used by young people and the use they make of them. In fact, this line of research would complete our literature review of the research question RQ1. Second, it is intended to define a methodology for evaluating the level of security and privacy of IoT devices aimed at minors. Finally, an analysis of the possible human factors that affect vulnerability and the risks detected is proposed.

## 7. Conclusions

This paper shows a complete vision of the IoT technological paradigm, focusing our study on minors between 12 and 17 years old. The main objective is to warn about vulnerabilities associated with the connected devices most used by the target group in their daily lives. In recent years, the development of the Information Society and the IoT has been booming, leading to the creation of ICT services focused on their personalized use by users. This issue is closely related to a high penetration of this ecosystem of interconnected devices in our daily lives, which has a significant impact on a personal level and in areas such as Smart Cities, Smart Homes, etc.

This study is of particular interest because, although the IoT benefits are clear, the set of devices that comprise it faces several vulnerabilities associated with its use. Therefore, we conducted a literature analysis on the types of connected devices most used by the target group, minors from 12 to 17 years old, in their daily lives.

With this information, we categorized these connected devices into eight interest groups: smartphones and tablets, smart TVs and game consoles, smart toys, wearables, smart home IoT devices, smart personal assistants, smart speakers, and others, such as web cameras, drones, or intimate devices.

After a detailed review of the literature, our research has shown numerous reports of worrisome security and privacy issues with these devices, so it is interesting to include them in the study. Following this categorization, we studied the main problems and security and privacy deficiencies associated with these devices. Specifically, most of these problems are driven by manufacturers neglecting security to lower the cost of their devices and non-technological users unaware of good security practices. The trend in this area is that as the years go by, users are becoming increasingly aware of the importance of their data. However, control measures have been put in place in the manufacturing processes to require manufacturers to comply with a minimum security of their devices, as well as regulations requiring the control of user data privacy. At the end of this study, we expose a clear view of the weaknesses found in each device category.

To analyze the problems associated with their use, we have developed a glossary of security and privacy issues for each device category. In this glossary, we propose seven principal security vulnerabilities and three privacy vulnerabilities, which are: (1) spoofing, (2) lack of or weak encryption, (3) lack of or weak authentication, (4) uncontrolled voice interaction, (5) code injection, (6) data interception and takeover, (7) regarding the security; and (8) user data being compromised, (9) violation of privacy laws and (10) lack of control and understanding, regarding the privacy. The set of vulnerabilities identified in the glossary covers all security and privacy areas that threaten attacks on devices.

On the one hand, the device security attacks identified through the literature review show that these are becoming increasingly sophisticated and personalized in search of specific end-user data. With respect to the privacy they offer, it is important to note that on many occasions users do not pay attention to the privacy policy they are accepting, either due to a lack of clarity in it or because they accept it without reading. This may imply a data collection, based on this consent, not always transparent to the end user. It is therefore consistent that only the necessary information required should be collected and be adequately protected, and this should be adequately and transparently notified to the user.

In fact, the clear relationship identified between the glossary’s security and privacy issues has been exposed. In this way, it makes sense to talk about security together with privacy and the need to search for strategies that allow the protection of security and privacy together. Thus, through a matrix, we provide a visual representation of the devices with the most security and privacy vulnerabilities and, therefore, especially vulnerable to attacks.

This research raises awareness of severe security vulnerabilities in connected devices used by young people and will help other researchers address these issues with a more global perspective. In addition, knowledge of such security and privacy issues in connected devices will enable users to be aware of the threats associated with the use of these devices and to learn good usage practices to avoid such attacks.

## Figures and Tables

**Table 1:** Glossary of the security and privacy issues defined.

Issue	ID	Term	Description
Security	(1)	Spoofing	Impersonation of the identity of the user or the device.
	(2)	Lack of or weak encryption	Exposure of data during the transfer of information between peers since these are exchanged in plain text or protected by unreliable or obsolete encryption methods.
	(3)	Lack of or weak authentication	Obsolete or null authentication mechanisms that allow access to the device with a specific role.
	(4)	Uncontrolled voice interaction	Possibility of execution of voice commands by strangers or unauthorized users, as well as side-channel attacks.
	(5)	Code injection	Execution of malicious commands prepared to modify the common operation of the system or facilitate unauthorized access to protected data.
	(6)	Data interception	Active or passive (sniffing) listening of communications between interconnected devices that goes unnoticed by common users.
	(7)	Takeover	Taking full control of the device to access data or carry out attacks that require cooperation between connected devices.
Privacy	(8)	User data being compromised	Operation of the connected device, the underlying server, or third-party applications involving the loss, misuse, or unauthorized user data.
	(9)	Violation of privacy laws	Improper use of sensitive and/or personal data that implies a total or partial violation of specific privacy laws such as the COPPA, etc.
	(10)	Lack of control and understanding	Loss of control over the management of user data and/or ignorance of the use made by the underlying devices and applications or services. This issue considers the user perception of what happens with the personal data managed by the device or underlying application.

**Table 2:** Traceability matrix about kind of the devices and privacy and security issues.

	Security Issues							Privacy Issues			
**Device Category**	**(1)**	**(2)**	**(3)**	**(4)**	**(5)**	**(6)**	**(7)**	**(8)**	**(9)**	**(10)**	**Refs.**
Smartphones and Tablets								Yes	Yes	Yes	[27,28,29]
Smart TVs and Game Consoles	Yes				Yes	Yes		Yes		Yes	[30,32,33,35,36,37]
Smart Toys		Yes	Yes				Yes	Yes	Yes		[39,40,41,42,43]
Wearables	Yes	Yes	Yes			Yes	Yes	Yes		Yes	[44,46,47,48,49,50,51]
Smart Home IoT Devices	Yes	Yes	Yes		Yes	Yes	Yes	Yes		Yes	[54,55,56,57,58,59,60]
Smart Personal Assistants	Yes		Yes	Yes				Yes		Yes	[61,62,63,64]
Smart Speakers	Yes			Yes	Yes	Yes	Yes	Yes		Yes	[67,68,69,70,71,72,73,74,75,76]
Others		Yes	Yes		Yes	Yes	Yes	Yes			[77,78,79,80]

Note: (1) Spoofing, (2) lack of or weak encryption, (3) lack of or weak authentication, (4) uncontrolled voice interaction, (5) code injection, (6) data interception, (7) takeover; (8) user data being compromised, (9) violation of privacy laws, (10) lack of control and understanding.

## Data Availability

Not applicable.
